# Apolipoprotein C-II and lipoprotein lipase show a temporal and geographic correlation with surfactant lipid synthesis in preparation for birth

**DOI:** 10.1186/1471-213X-10-111

**Published:** 2010-11-08

**Authors:** Mélissa Côté, Pierre R Provost, Marie-Christine Gérard-Hudon, Yves Tremblay

**Affiliations:** 1 Reproduction, Perinatal and Child Health Axis, Rm T-1-49, CHUQ Research Center; Centre de Recherche en Biologie de la Reproduction (CRBR), Laval University, Québec City, Québec, Canada; 2 Department of Obstetrics and Gynecology, Faculty of Medicine, Laval University, Québec City, Québec, Canada

## Abstract

**Background:**

Fatty acids are precursors in the synthesis of surfactant phospholipids. Recently, we showed expression of apolipoprotein C-II (apoC-II), the essential cofactor of lipoprotein lipase (LPL), in the fetal mouse lung and found the protein on the day of the surge of surfactant synthesis (gestation day 17.5) in secretory granule-like structures in the distal epithelium. In the present study, we will answer the following questions: Does apoC-II protein localization change according to the stage of lung development, thus according to the need in surfactant? Are LPL molecules translocated to the luminal surface of capillaries? Do the sites of apoC-II and LPL gene expression change according to the stage of lung development and to protein localization?

**Results:**

The present study investigated whether the sites of apoC-II and LPL mRNA and protein accumulation are regulated in the mouse lung between gestation day 15 and postnatal day 10. The major sites of apoC-II and LPL gene expression changed over time and were found mainly in the distal epithelium at the end of gestation but not after birth. Accumulation of apoC-II in secretory granule-like structures was not systematically observed, but was found in the distal epithelium only at the end of gestation and soon after birth, mainly in epithelia with no or small lumina. A noticeable increase in surfactant lipid content was measured before the end of gestation day 18, which correlates temporally with the presence of apoC-II in secretory granules in distal epithelium with no or small lumina but not with large lumina. LPL was detected in capillaries at all the developmental times studied.

**Conclusions:**

This study demonstrates that apoC-II and LPL mRNAs correlate temporally and geographically with surfactant lipid synthesis in preparation for birth and suggests that fatty acid recruitment from the circulation by apoC-II-activated LPL is regionally modulated by apoC-II secretion. We propose a model where apoC-II is retained in secretory granules in distal epithelial cells until the lumina reaches a minimum size, and is then secreted when the rate of surfactant production becomes optimal.

## Background

The preparation of the lung for an aerobic environment includes the surge of surfactant synthesis, which occurs late in pregnancy in Type II pneumocytes (PTII) in the distal epithelium (for reviews see [1,2]). Pulmonary surfactant is a combination of lipids and proteins [3,4] enabling normal respiration by preventing alveolar collapse. Surfactant deficiency is the major cause of respiratory distress syndrome of the neonate (or hyaline membrane disease) [5,6], a pathology occurring when birth arises before adequate PTII cell maturation.

Fatty acids are precursor molecules in the synthesis of surfactant phospholipids. They can be synthesized in the lung or originate from circulating triglycerides. In the plasma, triglycerides are mainly found in the core of VLDL and chylomicrons, the latter carrying alimentary lipids after secretion by the small intestine. In many tissues including adipose tissue and skeletal muscle, delivery of fatty acids from triglyceride-rich lipoproteins occurs by hydrolysis on the luminal surface of the capillary endothelium. This reaction is catalyzed by lipoprotein lipase (LPL) [7,8] and requires apolipoprotein C-II (apoC-II) as essential and specific cofactor [9,10].

LPL expression was studied in the mature lung. In the guinea pig, LPL mRNA was mainly found in alveolar macrophages, while the protein was mainly localized in capillaries [11]. This is compatible with a previous observation in the adult rat where LPL activity was found in lung macrophages [12]. In the human, fetal lung explants from the second trimester of gestation were studied [13]. LPL protein was found at the surface of epithelial cells after stimulation of the tissue with dexamethasone/8-Br-cAMP/isobutylmethylxanthine.

Recently, we reported expression of LPL and apoC-II in the fetal mouse lung between gestation days (GD) 15.5 to 18.5 [14]. A sex difference in the level of apoC-II mRNA was observed (P = 0.0195), while a significant increase in LPL mRNA was found from GD 17.5 to 18.5 (P = 0.0003). Immunohistochemistry (IHC) revealed the presence of apoC-II in secretory granule-like structures in the distal epithelium, mainly near the basal membrane, close to the mesenchyme, a structure that is distinct from lamellar bodies [14]. The fact that the apoC-II protein is found at this site on the day when the surge of surfactant synthesis occurs suggests the participation of apoC-II in this process. Many questions arise from this work for which an answer will clarify the role of apoC-II and LPL in surfactant synthesis. Does apoC-II protein localization change according to the stage of lung development, thus according to the need in surfactant? Are LPL molecules translocated to the luminal surface of capillaries? Do the sites of apoC-II and LPL gene expression change according to the stage of lung development and to protein localization? To answer these questions, we have performed in situ hybridization (ISH) and IHC of LPL and apoC-II from GD 15.5 to the first days of alveolarization. QPCR analysis of apoC-II and LPL mRNAs was also performed with samples from GD 19.5 to postnatal day 10 (PN 10).

## Results

### mRNA and protein localization of LPL and apoC-II during the pseudoglandular and the canalicular stages

The surge of surfactant synthesis occurs on GD 17.5 in the mouse as indicated by the appearance of lamellar bodies [15], an increase in surface activity in the mouse lung homogenate [15], and by increases in the activity of some enzymes involved in pulmonary lipid metabolism [16,17]. Therefore, we have first studied samples from pseudoglandular (GD 15.5) to late canalicular (GD 17.5) stages of lung development in order to determine whether mRNA and/or protein localization of LPL and apoC-II change in preparation to the surge of surfactant synthesis.

**Figure 1:**
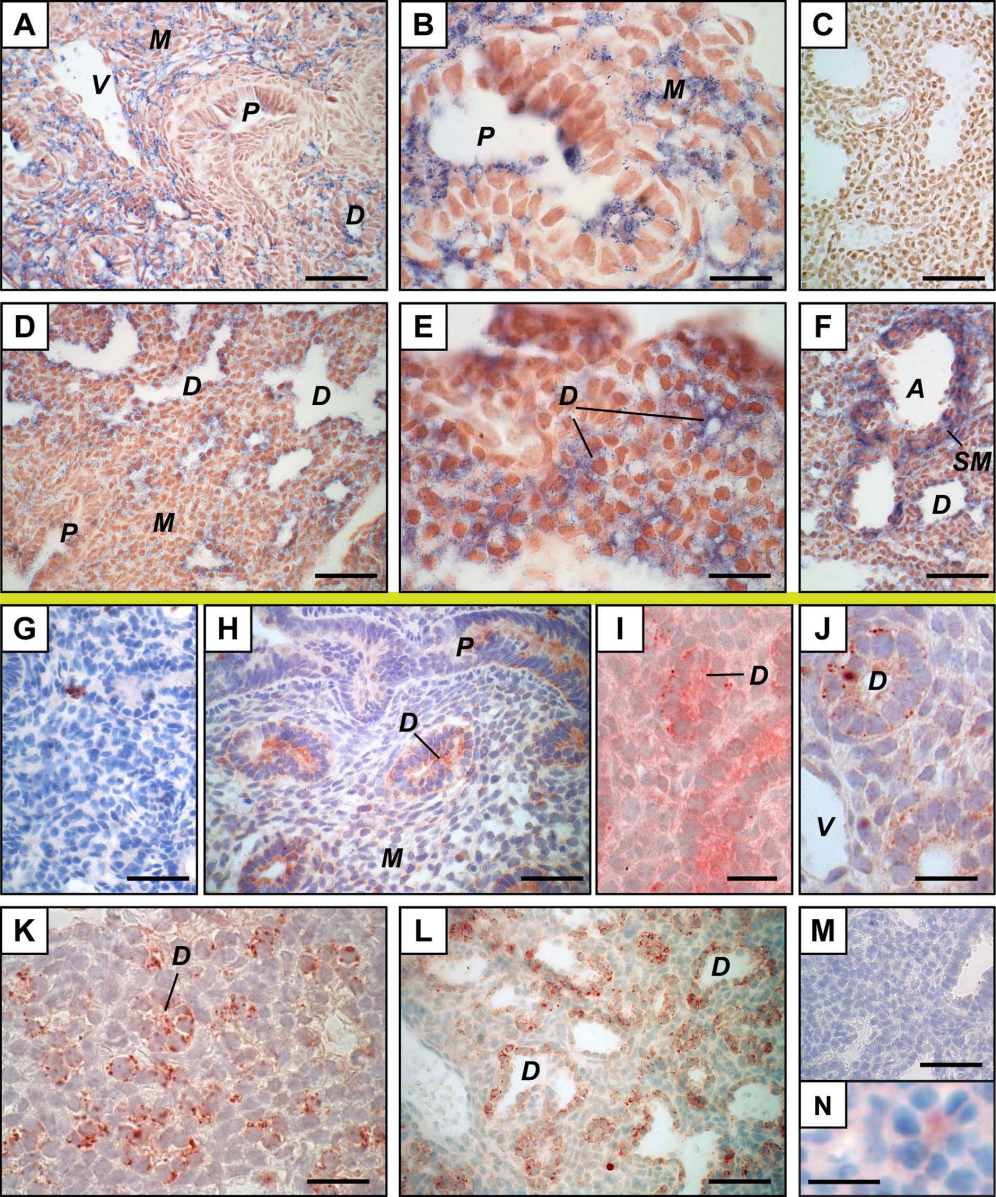
**Distribution of apolipoprotein C-II mRNA and protein in the mouse fetal lung**. Mouse tissue sections are from pseudoglandular (GD 15.5, A, B, G, H), junction between pseudoglandular and canalicular (GD 16.5, I, J), or late canalicular (GD 17.5, C to F, K to N). *In situ *hybridization (above the yellow line) (A to F) was performed with apoC-II anti-sense (A, B, D to F) and sense (C) probes. A change in sites of apoC-II mRNA synthesis (positive signal, blue) according to developmental time was observed. Immunohistochemistry (below the yellow line) (G-N) was performed using an anti-apoC-II polyclonal antibody (H to L), an anti-cytokeratin 18 monoclonal antibody (N), or goat IgG as negative control (G, M). Positive signal (red) was found in the distal epithelium from GD 15.5 to GD 17.5, but it was found in secretory granule-like structures during the canalicular stage, but not the pseudoglandular stage. A transition state was observed on GD 16.5 (I, J). Scale bars, 50 μm (A, C, D, F to H, L, M), 20 μm (B, E, I to K), or 10 μm (N). ***A***, artery; ***D***, distal epithelium; ***M***, mesenchyme; ***P***, proximal epithelium; ***SM***, smooth muscle; ***V***, vein.

On GD 15.5, apoC-II mRNA was found mainly in the mesenchyme (Figure 1A-B). Positive signals were also found in cells of some epithelia (Figure 1A-B), while the most proximal epithelium was negative (Figure 1A). In contrast, the apoC-II protein did not accumulate in all the sites of apoC-II gene expression on GD 15.5 but only in the epithelium where a diffuse signal was observed in the cytoplasm (Figure 1H). On GD 17.5, the apoC-II mRNA and protein co-localized. They were found only in the distal epithelium with lumina (Figure 1D, L) and the most distal epithelium before apparition of the lumina (Figure 1E, K). It should be noted that structures corresponding to the most distal epithelium differ between GD 15.5 and 17.5. The most distal epithelium observed on GD 15.5 is no longer the most distal epithelium on GD 17.5 where a more differentiated epithelium is observed. The identity of cells composing the most distal epithelium with no lumina on GD 17.5 was confirmed by IHC using an anti-cytokeratin 18 antibody (Figure 1N), which is a marker of epithelial cells [18].

Recently we reported that the apoC-II protein accumulated on GD 17.5 in the distal epithelium, more precisely in structures looking like secretory granules, mainly localized near the basal membrane, close to the mesenchyme [14]. Similar results were obtained here (Figure 1K). Interestingly, we show here that localization of the positive signal in the cells is different on GD 15.5 compared to GD 17.5. No positive secretory granules were observed on GD 15.5. Accordingly, data obtained on GD 16.5 show a transition state between results at GD 15.5 and those at GD 17.5. As shown in Figure 1I, both a diffuse positive signal in the cytoplasm of epithelial cells and a few dots looking like secretory granules were observed in the same tissue. Figure 1J is from another GD 16.5 litter and shows small positive dots, but no diffuse signal in the epithelium, which could represent a later developmental event. No positive signal was observed by ISH using lung materials of 6 fetuses from 3 litters sacrificed on GD 16.5 (data not shown), which is also compatible with a transition state.

**Figure 2:**
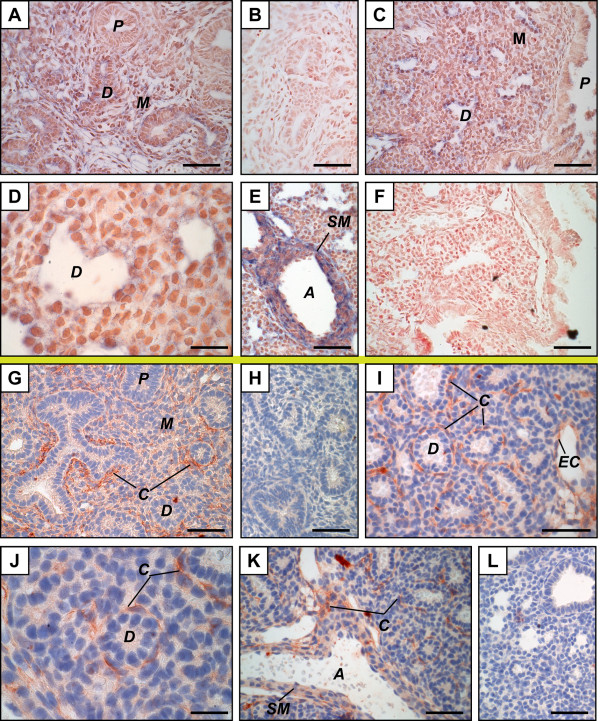
**Distribution of lipoprotein lipase mRNA and protein in the mouse fetal lung**. Mouse tissue sections are from pseudoglandular (GD 15.5, A, B, G, H) or late canalicular (GD 17.5, C to F, I to L) stages. *In situ *hybridization (above the yellow line) (A to F) was performed with LPL anti-sense (A, C to E) and sense (B, F) probes. The site of LPL mRNA synthesis (positive signal, blue) changed according to gestation time. Immunohistochemistry (below the yellow line) (G-L) was performed using an anti-LPL polyclonal antibody (G, I to K) or goat IgG as negative control (H, L). Positive signals (red) were mainly found on capillaries from GD 15.5 to 17.5. Scale bars, 50 μm (A to C, E to I, K, L) or 20 μm (D, J). ***A***, artery; ***C***, capillary; ***D***, distal epithelium; ***EC***, endothelial cell; ***M***, mesenchyme; ***P***, proximal epithelium; ***SM***, smooth muscle.

LPL protein was found in capillary-like structures on GD 15.5, GD 16.5, and GD 17.5 (Figure 2G-L and data not shown). LPL mRNA was mainly found in epithelial cells of the distal epithelium on GD 17.5 (Figure 2C-D). This mRNA was absent of the proximal epithelium on GD 17.5 except for a few cells that may present barely detectable signals (Figure 2C). On GD 15.5, LPL mRNA was mainly found in the mesenchyme and the distal epithelium (Figure 2A) as for apoC-II mRNA. On GD 16.5, no signal was obtained by ISH for LPL using lung tissues of 7 fetuses from 4 litters (data not shown).

**Figure 3:**
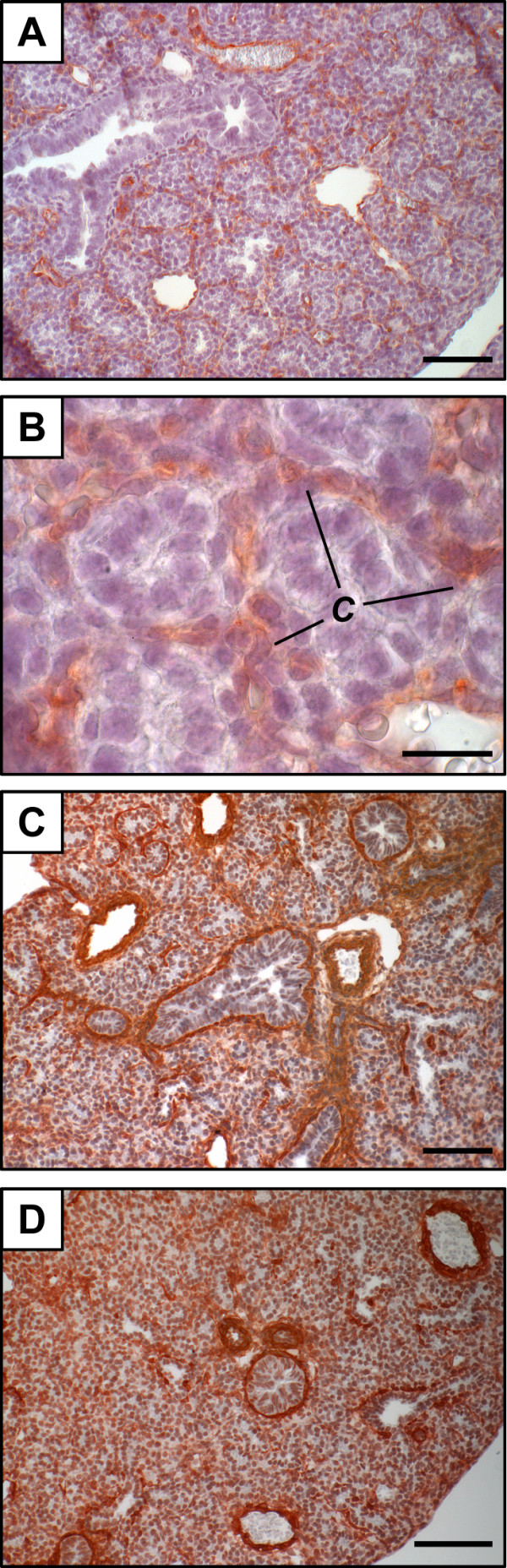
**Distribution of PECAM-1 and α-sma proteins in the mouse fetal lung**. Mouse tissue sections isolated on GD 17.5 were subjected to immunohistochemistry using anti-PECAM-1 (A, B) or anti-α-sma (C, D) as primary antibodies. Capillaries are PECAM-1-positive. Specific staining is clearly different between the two antibodies and only the staining pattern obtained with the anti-PECAM-1 corresponds to positive signals obtained with the anti-LPL antibody in Figure 2. Scale bars, 80 μm (A, C), 20 μm (B) or 40 μm (D). ***C***, capillaries.

In order to confirm that the LPL-positive signal corresponds to capillaries and not to α-smooth muscle actin (α-sma)-positive structures, an anti-platelet endothelial cell adhesion molecule-1 (PECAM-1) antibody was used on GD 17.5-lung tissue sections to stain capillaries. Results show that staining of capillary network (Figure 3A-B) is very similar to the positive signals identified as capillary-like structures using an anti-LPL antibody. In contrast, the use of an anti-α-sma antibody leads to a different staining profile (Figure 3C-D).

ApoC-II (Figure 1F) and LPL (Figure 2E) mRNAs were observed in smooth muscles surrounding large arteries. LPL protein was found in smooth muscles of arteries (Figure 2K), but signal intensities were lower than those found in adjacent capillaries. No IHC signal was found in smooth muscles for apoC-II (data not shown). In most cases, it was difficult to determine whether endothelial cells of large vessels were stained because of the proximity to positive structures. Nevertheless, endothelial cells positive for LPL by IHC (Figure 2I) and others negative for apoC-II by ISH and IHC (data not shown) were also found on GD 17.5.

### ApoC-II and LPL mRNA levels during the saccular stage and the first segment of the alveolar stage

**Figure 4:**
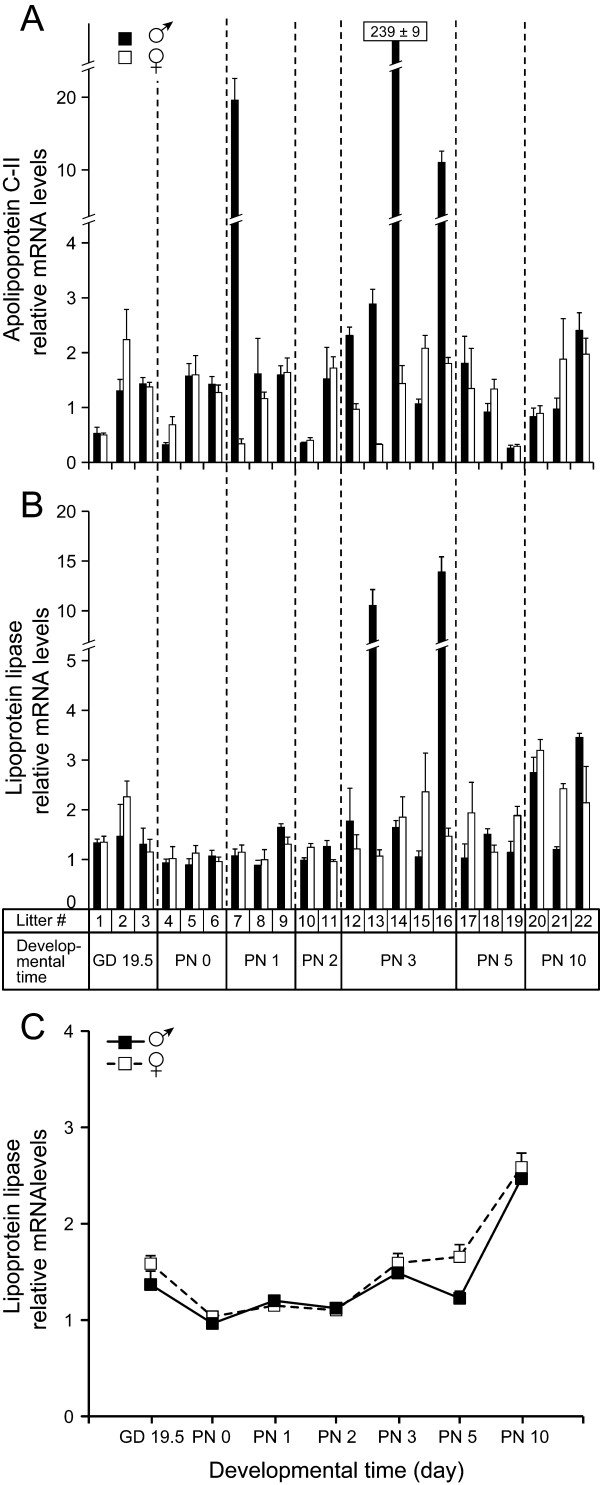
**Apolipoprotein C-II and lipoprotein lipase mRNA levels in perinatal mouse lungs**. Expression of apoC-II (A,) and lipoprotein lipase (B, C) genes in developing lungs of male and female mouse fetuses of several litters between GD 19.5 and PN 10. (A and B): Each bar represents the value (± S.D.) obtained from technical duplicate using a pool of male or female fetuses of the indicated litter. (C): Means ± SEM of relative mRNA levels according to developmental time (day). The values were calculated from the results presented in B excluding the outlier values obtained for males of litters no. 13 and 16. The values were normalized by two housekeeping genes. For apoC-II and LPL, the mean values respectively obtained at PN 2 and PN 0 were fixed as onefold.

ApoC-II and LPL QPCR data were recently published for samples from GD 15.5 to GD 18.5 [14], but not for further developmental times. Here, QPCR analysis of these two genes is presented between GD 19.5 and PN 10 using male and female pools of various litters (Figure 4). Levels of apoC-II mRNA showed more variations from sample to sample than those of LPL mRNA. There was no statistically significant sex difference for the two genes, even though higher values were observed for males for apoC-II mRNA on PN 3 in four of the five analyzed litters (Figure 4A). A statistically significant difference according to developmental time was observed only for LPL mRNA (*P *= 0.0005) (Figure 4C).

### mRNA and protein localization of LPL and apoC-II during the saccular and the beginning of the alveolar stages of lung development

**Figure 5:**
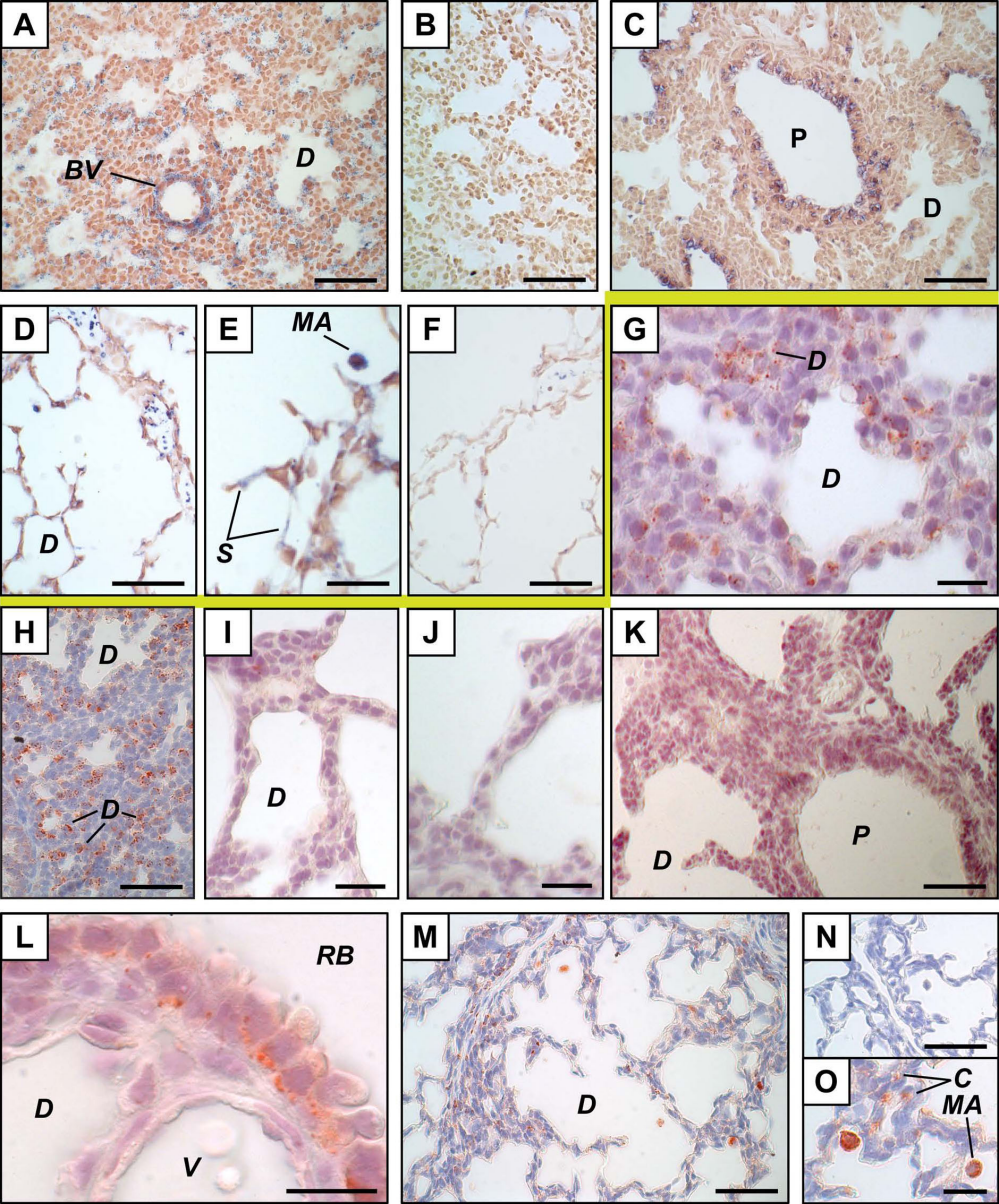
**Distribution of apolipoprotein C-II mRNA and protein in the perinatal mouse lung**. Mouse tissue sections are from saccular stage (A, B, H, GD 19.5; C, G, PN 0; I, J, PN 1; K, PN 2; L, PN 3; D to F, M, N, PN 5) or alveolar stage (O, PN 10). *In situ *hybridization (above the yellow line) (A to F) was performed with apoC-II anti-sense (A, C to E) and sense (B, F) probes. The major site of apoC-II mRNA synthesis (positive signal, blue) changed after birth (compare A to C). Positive signals were found in newly-formed septa (D, E) and macrophages (E) on PN 5. Immunohistochemistry (below the yellow line) (G-O) was performed using an anti-apoC-II polyclonal antibody (G to I, K to M, O) or goat IgG as negative control (J, N). Positive signals (red) were found in secretory granule-like structures in distal epithelial cells on GD 19.5 (H) and PN0 (G) but not in later timepoints. On PN 3, positive secretory granules were also found in epithelial cells of the respiratory bronchioles near the basal membrane, close to the mesenchyme (L). Macrophages were positive on PN 5 and 10 while capillaries were positive on PN 10 in one third of the analyzed subjects (O and data not shown). Scale bars, 50 μm (A to D, F, H, K, L, M, N) or 20 μm (E, G, I, J, L, O). ***BV***, blood vessel; ***C***, capillary; ***D***, distal epithelium; ***MA***, macrophage; ***P***, proximal epithelium; ***RB***, respiratory bronchiole; ***S***, septa; ***V***, vein.

**Figure 6:**
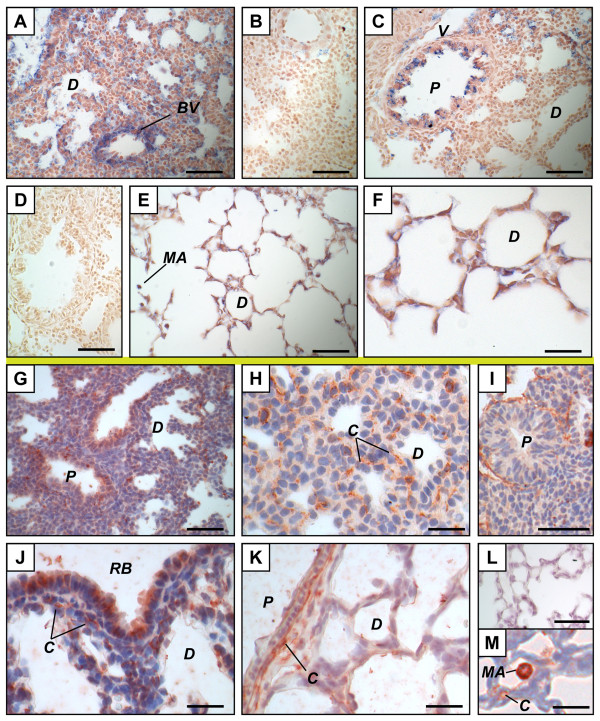
**Distribution of lipoprotein lipase mRNA and protein in the perinatal mouse lung**. Mouse tissue sections are from saccular stage (A, B, GD 19.5; C, D, G, H, PN 0; I, PN 3; E, F, J, K, PN 5) or alveolar stage (L, M, PN 10). *In situ *hybridization (above the yellow line) (A to F) was performed with LPL anti-sense (A, C, E, F) and sense (B, D) probes. The major site of LPL mRNA synthesis (positive signal, blue) changed at birth (compare A and C). Positive signals were found in the alveolar wall and possibly in macrophages on PN 5. Immunohistochemistry (below the yellow line) (G-M) was performed using an anti-LPL polyclonal antibody (G to K, M) or goat IgG as negative control (L). Positive signals (red) were found in capillaries in all the analyzed developmental times, in respiratory bronchioles from GD 19.5 to PN 3 (J, K and data not shown), and in macrophages on PN 5 and PN 10 (M and data not shown). Scale bars, 50 μm (A to E, G, L) or 20 μm (F, H to K, M). ***BV***, blood vessel; ***C***, capillary; ***D***, distal epithelium; ***MA***, macrophage; ***P***, proximal epithelium; ***RB***, respiratory bronchiole; ***V***, vein.

Similarities were found between apoC-II and LPL expression patterns during the perinatal period. Both genes were expressed in the distal epithelium short time before birth (Figures 5A and 6A), but not after birth (Figures 5C and 6C) (delivery occurred during GD 19). In contrast, the proximal epithelium was negative on GD 17.5, showed a weak positive signal on GD 18.5 and 19.5 (data not shown), presented a marked increase in intensity soon after birth (Figures 5C and 6C), and was negative on PN 5 for both genes. Using 6 fetuses on GD 19.5 (3 males and 3 females) and 6 neonates on PN 0, we confirmed that the switch from the distal to the proximal epithelium correlated with birth, not with sex (data not shown). ApoC-II and LPL mRNAs were also found in a few scattered distal epithelial cells after birth. In addition, positive signals by ISH were observed in association with blood vessels before birth (Figures 5A and 6A) but not after birth (data not shown) for both genes. On PN 5, thus at junction between saccularization and alveolarization, apoC-II and LPL mRNA were found in alveolar walls (Figures 5D-E and 6E-F), including newly-formed septa (Figure 5E and data not shown).

**Figure 7:**
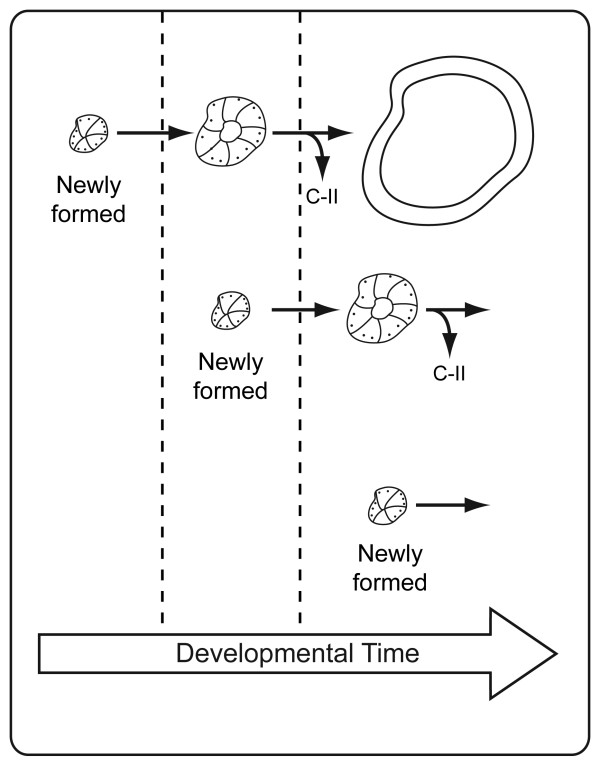
**Model of apoC-II secretion**. Dotted lines separate different developmental time points and, for each time point, the presented distal epithelial structures are co-observed in the same lung tissue sections. ApoC-II-containing secretory granules (dots) are observed in distal epithelia with no or small lumina from GD 16.5 to PN 0, but not in the distal epithelia with a large lumina between GD 18.5 and PN 0. These observations suggest that apoC-II secretion is delayed until apparition of the lumina. After its secretion, apoC-II can reach LPL molecules localized on the luminal surface of capillaries to activate LPL activity and fatty acid recruitment. According to this hypothesis, it could be difficult to detect apoC-II after its secretion because of the nature of its association with the tissue.

Albeit similarities were observed in the expression profiles of their encoding genes, apoC-II and LPL proteins did not accumulate in the same structures. Interestingly, apoC-II was observed in secretory granule-like structures until soon after birth (Figure 5G and data not shown), but positive signal had disappeared one day later (Figure 5I). In fact, between GD 18.5 and PN 0, apoC-II-positive secretory granules were mainly observed in distal epithelia with no or small lumina, but not with large lumina (Figure 5G-H and data not shown). Such a discrepancy was less evident for apoC-II mRNA for which some distal epithelia with large lumina were also positive (Figure 5A). This strongly suggests that apoC-II protein is synthesized and retained within the epithelium in secretory granules until the lumina reaches a certain diameter, and that it is then secreted (Figure 7). We did not observe any sex difference or developmental delay in our experiment in that the three males and the three females analyzed on PN 0 presented secretory granules, while the six other animals studied on PN 1 did not show any secretory granule (data not shown). In all of the experiments, neonates sacrificed on PN 0 were killed between a few minutes and four hours after the birth of the first neonate. The apoC-II protein was still undetectable on PN 2 (Figure 5K), while secretory granules were found in the epithelial cells of the respiratory bronchioles on PN 3, near the basal membrane, close to the mesenchyme (Figure 5L). Only a few secretory granules were still positive on PN 5. ApoC-II-positive capillaries, as those shown on Figure 5O, were only observed in one third of the subjects analyzed on PN 10. In fact, after its secretion, apoC-II protein may be difficult to detect depending of the nature of its association with the tissue.

The situation was clearly different for LPL. The protein was found in capillaries from PN 0 to PN 10 for all the analyzed neonates (Figure 6 H-K, M, and data not shown) as well as before birth. LPL positive signals were also found in cells of the respiratory bronchiole from GD 19.5 to PN 3 (Figure 6G-J and data not shown), but not on GD 18.5 and PN 5. In this epithelium, signal intensity showed variation from cell to cell. In contrast, no protein was detected in most proximal epithelium (Figure 6I).

Cells localized in the alveolar space on PN 5 were positive for apoC-II and LPL by both ISH (Figures 5E and 6E) and IHC (data not shown) with no sex difference. Based on their localization and morphology, these cells were most probably macrophages. ApoC-II and LPL proteins were also found in macrophages on PN 10 (Figures 5O and 6M).

### Disaturated phosphatidylcholine determination

**Figure 8:**
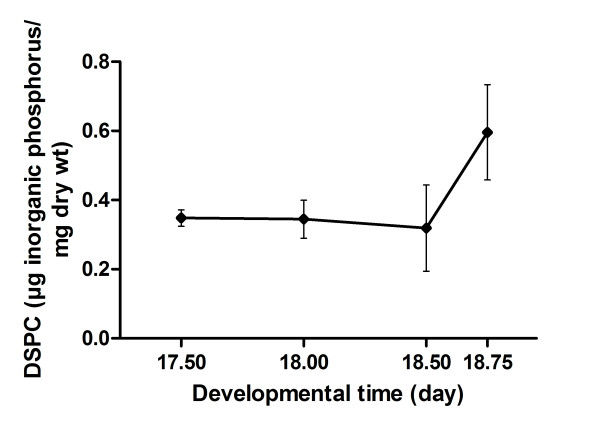
**Production of surfactant DSPC in the developing lung of Balb/C mice**. Relative DSPC content (in μg inorganic phosphorus/mg dry wt) in Balb/C fetal lungs according to developmental time (day). A one-hour mating window was used (± 0.02 day). Within the developmental time window studied, DSPC varied significantly (P < 0.0001, one-way ANOVA) between GD 18.50 and 18.75 (P < 0.05, Tukey's multiple comparison test).

Disaturated phosphatidylcholine (DSPC) is quantitatively the most important lipid of surfactant [19] and an increase in lung DSPC content was observed after the surge of surfactant synthesis [20]. Here, relative DSPC levels were determined in lungs from Balb/C fetal mice from GD 17.50 to 18.75 following a one hour mating window (GD ± 0.02). A marked statistically significant increase was observed between GD 18.50 and GD 18.75 (Figure 8).

## Discussion

Surfactant is of the first importance in the distal epithelium to prevent alveolar collapse. Our results show that the LPL-related molecular machinery is regulated temporally and geographically in the developing lung in correlation with the need in surfactant lipid synthesis. Major changes in sites of apoC-II and LPL mRNA are summarized in Table 1. Changes in protein localization were mainly observed for apoC-II and are summarized in Table 2. Our results show that the tissue needing surfactant governs itself LPL and apoC-II gene expression and apoC-II secretion during the critical period. Thus, the LPL machinery is subjected to a complex regulation during the canalicular and the saccular stages of lung development. The need of lipids for surfactant synthesis is particularly important during the period between the surge of surfactant synthesis and birth. This can explain why the distal epithelium of the lung contributes itself to LPL and apoC-II synthesis, which must maximizes fatty acid recruitment during this period. Our results are compatible with those of Ryan et al [21] showing that maternal loading with VLDL stimulates fetal surfactant synthesis.

**Table 1:** Major sites of apoC-II and LPL mRNA accumulation

	GD 15.5	GD 17.5	GD 18.5	GD 19.5	PN 0	PN 5
Mesenchyme	CII & LPL					
Distal epithelium	CII & LPL	CII & LPL	CII & LPL	CII & LPL		
Respiratory bronchioles					CII & LPL	
Septa						CII & LPL
Macrophages						CII & LPL

**Table 2:** Major sites of apoC-II and LPL protein accumulation

	GD 15.5	GD 17.5	GD 18.5	GD 19.5	PN0	PN1	PN2	PN3	PN5	PN10
Epithelium	C-II									
Secretory granules-distal epithelium										
- with no or small lumina		C-II	C-II	C-II	C-II					
- with large lumina		C-II								
Secretory granules-respiratory bronchioles								C-II		
Respiratory bronchioles				LPL	LPL	LPL	LPL	LPL		
Capillaries	LPL	LPL	LPL	LPL	LPL	LPL	LPL	LPL	LPL	LPL (CII)*
Macrophages									CII & LPL	CII & LPL
Not observed						C-II	C-II			

* ApoC-II was observed in capillaries only in one third of the analyzed subjects on PN 10

One major observation about apoC-II protein is that it accumulated in secretory granules within several distal epithelia from GD 17.5 to a developmental time point soon after birth. Thus, a temporal and geographic correlation exists between the appearance of lamellar bodies as reported in the literature [15] and apoC-II accumulation in secretory granules presented in Figure 1. In contrast to apoC-II, LPL protein was obviously secreted out of the producing cells and accumulated in capillaries. Accordingly, a rapid release of 10-15% of total tissue LPL was observed following infusion of heparin into isolated perfused adult rat lungs ([22] and references therein). The presence of LPL in capillaries in the fetal lung is compatible with its recognized function in recruitment of fatty acids from lipoprotein-associated triglycerides [23,24]. In contrast to LPL, apoC-II was observed in its producing cells within secretory granules. This suggests that apoC-II secretion is controlled, but not at all developmental time points. For example, no secretory granule is observed on GD 15.5 indicating that there is no apoC-II retention within the producing cells. A transition state is observed on GD 16.5 with small secretory granules, which must correspond to the retention of newly formed apoC-II molecules. From GD 18.5 to PN 0, distal epithelia with no or small lumina containing apoC-II-positive secretory granules as well as distal epithelia with large lumina negative for apoC-II were observed. As explained in Figure 7, these observations suggest that apoC-II is secreted by distal epithelia reaching a specific level of development, which stimulates local LPL activity for fatty acid recruitment for surfactant synthesis. In contrast to LPL, apoC-II was not reported to bind to the endothelium and consequently, most of the apoC-II molecules secreted by the epithelia would be washed off of the slide during the IHC procedure so that the presence of apoC-II in capillaries could be difficult to demonstrate. The fact that apoC-II is produced by the lung and not only provided by plasma for activation of LPL in capillaries could be explained by the great need for fatty acids for surfactant production and, consequently, the very high amount of LPL molecules necessitating apoC-II for their activation.

A noticeable increase in surfactant lipid content was observed between GD 18.50 and 18.75 (Figure 8). Knowing that surfactant lipids cannot be detected immediately when they start to be produced but have to accumulate over the detection limit before an increase be observed, our results are compatible with the literature showing the appearance of lamellar bodies on GD 17.5 [15]. Moreover, the marked increase in DSPC levels coincides with the presence of distal epithelium with large lumina without secretory granules, thus after the beginning of apoC-II secretion for optimization of surfactant lipid production based on our model (Figure 7).

In 1998, LPL expression was found in lipofibroblasts isolated from rat developing lungs [25]. More specifically, 20 fold and 3 fold increases in LPL mRNA levels were observed by Northern blot analysis in isolated lipofibroblasts and whole lungs, respectively, between postnatal days -3 and +2. In our experiments, not only did we fail to reproduce this rise at birth in whole mouse lungs by quantitative real time PCR, but LPL mRNA accumulation sites changed from distal to proximal epithelium at birth with no or only a few positive signal in the mesenchyme after birth. Furthermore, the IHC signal we obtained for LPL was close to the background in the mesenchyme during the perinatal period. Of course, it is not excluded that LPL expression in lipofibroblasts be very low and under the limit of detection in our assays, but obviously, our data indicate that this cell population is not a major site of LPL mRNA and protein accumulation. The results of Chen et al are also incompatible with other studies reporting many years ago that pulmonary LPL activity in growing rats peaks one day before birth and decreases at birth [26,27]. The decrease at birth may correspond to the switch we observed in the site of LPL gene expression in the mouse. In fact, the timing of LPL activity in the rats, the temporal and cell specific regulations of LPL gene expression we observed in the mouse and the presence of LPL in capillaries underscore a role in recruitment of lipids for the surge of surfactant synthesis. Such a role has been already proposed based on the presence of VLDL in the fetal circulation at 21 days' gestation in the rat when the peak in LPL activity occurs ([22] and references therein).

LPL proteins were also found in other sites in the developing lung as in respiratory bronchioles during saccularization and in macrophages from the beginning of alveolarization. As reported for rats, an increase in LPL activity was observed after the first week of life following the decrease at birth [26]. This observation is compatible with the increase we observed in LPL mRNA from PN 5 to 10 (Figure 4) and may be explained by apparition of intra-alveolar macrophages, which express LPL as reported here (Figure 6) and in the literature [12]. Our data cannot support or exclude LPL expression by lipofibroblasts at this developmental time.

No sex difference was observed in sites of apoC-II and LPL gene expression and protein accumulation for any of the analyzed time point. No sex difference in signal intensity was observed, although ISH and IHC are not reliable quantitative tools. QPCR analysis (Figure 4) did not reveal any significant sex difference when all the population was considered, but some differences from sample to sample prevented us to conclude at the absence of any sex difference at specific time points, as at PN 3 when higher levels of apoC-II mRNA were observed more frequently in males. The sex difference reported recently [14] in apoC-II mRNA levels in favor of females in developmental times preceding GD 19.5 is not ruled out by the present work. This statistically significant sex difference in apoC-II mRNA suggests that a significant proportion of females would start accumulating apoC-II earlier than males.

Hypertriglyceridemia is observed in both LPL and apoC-II deficiencies [8], while there is no evidence that these deficiencies may be associated with a high rate of mortality related to respiratory distress syndrome of the neonate in the human. The situation is different in LPL knockout mice, which present a high rate of neonatal death caused by respiratory problems [28]. However, the cause of death was engorgement of capillaries by chylomicrons leading to cyanosis, not surfactant insufficiency. Therefore, apoC-II and LPL seem not essential for obtaining minimal surfactant levels following birth at term. This situation is not surprising knowing that LPL activity is only one of the two sources of fatty acids, the remaining source being de novo synthesis through fatty acid synthase. As it is frequently observed for essential mechanisms/metabolisms, a redundancy is observed here in the source of fatty acids for surfactant synthesis. Interestingly, in mice deficient in adipose tissue LPL, fat mass was preserved by large increases in endogenous fatty acid biosynthesis, which compensated for the loss of LPL activity [8,29]. A similar situation should occur in the developing lung of LPL- and apoC-II-deficient subjects. In fact, the importance of the work presented here lies in the fact that preterm birth frequently leads to surfactant insufficiency and therefore, apoC-II/LPL may become an interesting pharmaceutical target in that context.

## Conclusions

We have shown that sites of apoC-II as well as LPL mRNA and protein accumulation are regulated during the canalicular and the saccular stages of lung development. These changes show a complex regulation of the LPL molecular machinery during an important period for the adaptation of the lung to gas exchange. From this work, apoC-II appears to be an interesting potential pharmaceutical target for treatment and/or prevention of respiratory distress syndrome of the neonate.

## Methods

### Mouse tissue preparation

Protocols were approved by the Animal Care and Use Committee and the Institutional Review Board of the Centre de Recherche du Centre Hospitalier Universitaire de Québec (protocols no. 2005-091 and 2008-071-2). Except for DSPC determination, female and male Balb/c mice (Charles River Laboratories St-Constant S.A., St-Constant, Qc, Canada) were mated during the night (mating window ± 8 h). The day of copulatory plug was considered as GD 0.5 (term GD 19.5) while the beginning of PN 0 corresponded to delivery. For DSPC determination, a mating window of one hour was used and the middle of this window was considered as GD 0.00. Pregnant females were killed by exposure to a CO_2 _atmosphere. The fetal sex was identified by examination of the genital tract. Confirmation of individual sex was done by PCR amplification of the *Sry *gene. Fetal lungs were collected and either kept frozen until RNA extraction or fixed in 4% buffered paraformaldehyde for 48 h at 4°C. Tissues were paraffin-embedded and cut in 5 μm slices. ISH and IHC were performed on samples from one female and one male of three or four litters for each gestation or postnatal day studied.

### RNA probes and in situ hybridization

Specific amplicons were synthesized from fetal lung cDNA using oligonucleotides designed to span at least one intron. Amplified gene/GenBank accession number/position of the amplified sequence/5' oligonucleotide/3' oligonucleotide (sequences include one restriction site for sub-cloning): *Lpl*/[GenBank:NM_008509]/750-955/GGGGAATTC-CAGCTGGGCCTAACTTTGAG/GGGAAGCTT-AATCACACGGATGGCTTCTC; *ApoC-II*/[GenBank:NM_009695]/269-508/GGGGAATTC-CCTGGCTCTATTCCTGGTCA/GGGAAGCTT-AAAATGCCTGCGTAAGTGCT. These amplicons were cloned into pGEM-4Z (Promega Corp., WI, USA). DNA matrix for SP6 and T7 polymerases were prepared by PCR amplification of each of the subcloned amplicon with the oligonucleotides GGATTTAGGTGACACTATAGAATA and TAATACGACTCACTATAGGGAGAC, which overlap the 5' end of the SP6 and the T7 promoters, respectively. Then, RNA probes were prepared using digoxigenin (DIG)-UTP substrate (Roche Diagnostics, Qc, Canada) and SP6 (sense) or T7 (antisense) RNA polymerases (Roche Diagnostics), as previously described [30]. ISH was performed as reported [30] except that denatured DIG-cRNA probes were used at 5 ng/μl. Slides were counterstained with 0.25% neutral red.

### Immunohistochemistry

Tissues were deparaffinized and subjected to IHC as reported [30]. The anti-apoC-II (T-12) (1:100) and the anti-LPL (C-20) (1:20) antibodies were purchased from Santa Cruz Biotechnology Inc. (CA, USA). A mouse anti-cytokeratin 18 monoclonal antibody (Abcam Inc., MA, USA) (1:200) was also used. A goat IgG preparation (Vector Laboratories Inc, ON, Canada) was used instead of primary antibody as negative control. A mouse anti-α-sma (VWR International CO., QC, Canada) (1:200) was also used. A biotinylated donkey anti-goat IgG (Millipore Canada Ltd, ON, Canada) and a biotinylated goat anti-mouse IgG (Cedarlane, ON, Canada) were used as secondary antibodies. The signals were revealed with the streptavidin-biotin peroxidase reaction method using an ABC Vectastain elite kit (Vector Laboratories Inc) and 3-amino-9-ethylcarbazole (AEC, Sigma-Aldrich) as chromagen. Slides were counterstained with Mayer's hematoxylin.

Slightly different conditions were used for the rabbit anti-PECAM-1 antibody (Abcam Inc, Cambridge, MA, USA). After deparaffinization, the antigen retrieval step consisted in a treatment with proteinase K (10 mg/ml in PBS) for 20 min. Incubation with the primary (anti-PECAM-1 antibody (1:10)) and the secondary antibodies and all washing steps were performed in 0.1% Tween 20 in PBS.

### Quantitative real-time PCR and statistical analysis

RNA extraction, cDNA synthesis, and QPCR were performed as described [14]. QPCR values were normalized for the amount of RNA input for each sample using a set of normalization factors obtained as described [14,31] from glyceraldehyde-3-phosphate dehydrogenase (Gapdh) and hydroxymethylbilane synthase (Hmbs) housekeeping genes.

Statistical analysis was performed using all data, including the outlier values. To ensure normality and homogeneous variances of the data set, the natural logarithm and the reciprocal of the dependent variable (expression) were used for apoC-II and LPL, respectively. To study the effects of the day of gestation and sex parameters on the expression of each gene, two-way ANOVA with repeated measures analyses were conducted using GraphPad Prism 5.01 (GraphPad Software Inc., CA, USA). The day of gestation was considered as a fixed factor. To account for possible correlations between observations made from the same litter, male and female values were considered as repeated measures.

### DSPC determination and statistical analysis

DSPC determination was performed for each fetus of the 3 to 5 litters per time point. A small piece of frozen lung was used to determine wet weight to dry weight ratio. Each individual remaining fetal lung sample was weighed. Homogenization and lipid extraction were performed according to the method of Bligh and Dyer [32]. DSPC was then isolated as reported [33] using osmium tetroxide (Sigma-Aldrich, St Louis, MO, USA) and a column of aluminum oxide (neutral alumina, activated, 150 mesh, Sigma-Aldrich). The resulting DSPC molecules were hydrolyzed to liberate phosphorus according to the method of Bartlett [34] with slight modifications. Briefly, 150 μl of 10N sulphuric acid was added and samples were heated at 150°C in a Pasteur oven for 2 hours. Then 50 μl of 30% phosphorus-free hydrogen peroxide were added before 24-hour incubation at 150°C. The amount of phosphorus corresponded to the amount of DSPC. Phosphorus assay was performed as follow [34]. A standard curve was made using a phosphorus standard solution (1000 μg/ml of P in H_2_O, Sigma-Aldrich). Fifty μl of 5% (w/v) ammonium molybdate and 50 μl Fiske-Subbarow reagent (15% Fiske-Subbarow Reducer, Sigma-Aldrich) were added. Samples were heated 20 min in a boiling water bath and the color, directly proportional to the concentration of inorganic phosphorus, was read at 825 nm in a Beckmann DU-640 spectrophotometer.

Statistical analyses were done using values of individual fetuses (10 to 25 individuals from 3 to 5 litters per developmental time point). One-way ANOVA was done to study the effect of developmental time on the amount of DSPC, followed by Tukey's multiple comparison test, both using GraphPad Prism software (version 5.01 for Windows, San Diego, CA). For all the analyses, a P-value < 0.05 was considered significant.

## List of abbreviations used

α-sma: α-smooth muscle actin; apoC-II: apolipoprotein C-II; DIG: digoxigenin; DSPC: disaturated phosphatidylcholine; GD: gestation day; IHC: immunohistochemistry; ISH: in situ hybridization; LPL: lipoprotein lipase; PECAM-1: platelet endothelial cell adhesion molecule-1; PN: post-natal day; PTII: type II pneumonocyte.

## Authors' contributions

MC carried out all laboratory manipulations except DSPC determination and contributed to interpretation of the data. M-C G-H carried out DSPC determination and the related 1-hour mating protocol. PRP and YT together conceived the project and analyzed the data. PRP supervised MC and wrote the manuscript. YT obtained funding and supervised the entire project. All the authors read and approved the final manuscript.
